# Supplementary material for: Forces and symmetry breaking of a living meso-swimmer
Source: Commun Phys. 2026 Jan 8;9(1):53. doi: 10.1038/s42005-025-02486-3 (PMC12893912; doi:10.1038/s42005-025-02486-3)
Supplement: Supplementary file 1 — Supplemenatary Materials [file 42005_2025_2486_MOESM1_ESM.pdf]

Supplementary Materials for

Forces and symmetry breaking of a living meso-swimmer

R. A. Lara[‡], N. Sharadhi[‡], A. A. L. Huttunen, L. Ansas, E. J. G. Rislakki, G. M. Bessa, and M. Backholm*
Department of Applied Physics, Aalto University, Espoo, Finland

*Email: matilda.backholm@aalto.fi; [‡]These authors contributed equally.

Supplementary Note 1: Calculations of the cantilever mass and damping coefficient

To confirm the accuracy of our dynamic calibration approach, we make order-of-magnitude calculations of the calibration outputs. We consider a typical cantilever with a length of $l_{\mathrm{MFS}} \approx 2$ cm and a tapered shape with minimum and maximum outer radii of $R_{\mathrm{o,min}} \approx 20$ µm and $R_{\mathrm{o,max}} \approx 50$ µm. From the dynamic calibration, we have measured $m_{\mathrm{eff}} \approx 10^{-7}$ kg and $b \approx 4 \cdot 10^{-5}$ Ns/m for such a cantilever (**Fig. 2D**). Given the complicated tapered geometry and tethered oscillations of the cantilever, it is not easy to calculate the exact values of its effective mass and damping coefficient. We thus simplify the cantilever as a straight cylinder with an average outer radius of $R_{\mathrm{o}} \approx 25$ µm and $R_{\mathrm{i}}/R_{\mathrm{o}} \approx 0.75$. The density of borosilicate glass and water is $\rho_{\mathrm{g}} = 2230$ kg/m^3 and $\rho_{\mathrm{w}} = 1000$ kg/m^3. This gives a cantilever mass of $m = \rho_{\mathrm{g}} l_{\mathrm{MFS}} \pi (R_{\mathrm{o}}^2 - R_{\mathrm{i}}^2) + \rho_{\mathrm{w}} l_{\mathrm{MFS}} \pi R_{\mathrm{i}}^2 = 6 \cdot 10^{-8}$ kg $\approx 10^{-7}$ kg. This is very similar to the measured effective mass ($m_{\mathrm{eff}} \approx 10^{-7}$ kg), which also includes an additional mass component of the external fluid moving with the cantilever.

For the damping coefficient, we assume a straight cylinder moving orthogonally through the fluid at low Re and use the Lamb equation for the drag coefficient $C_{\mathrm{d}} = 8\pi/[\mathrm{Re} \log(7.3/\mathrm{Re})]$. The drag force is $F_{\mathrm{d}} = 0.5\rho_{\mathrm{w}} U^2 C_{\mathrm{d}} S = 0.5\rho_{\mathrm{w}} U^2 l_{\mathrm{MFS}} \cdot 2R_{\mathrm{o}} \cdot 8\pi/[\mathrm{Re} \log(7.3/\mathrm{Re})] = 4\pi l_{\mathrm{MFS}} \eta U / \log\left(\frac{7.3\eta}{2R_{\mathrm{o}} \rho_{\mathrm{w}} U}\right) \equiv bU$, where the drag coefficient matching out damped hamronic oscillation model is $b = 4\pi l_{\mathrm{MFS}} \eta / \log\left(\frac{7.3\eta}{2R_{\mathrm{o}} \rho_{\mathrm{w}} U}\right) = 5 \cdot 10^{-5}$ Ns/m, using a cantilever speed of $U = 1$ mm/s which is the typical order of magnitude in the dynamic calibration experiments. Also, this result is in very good agreement with the measured value ($b \approx 4 \cdot 10^{-5}$ Ns/m).

The above rough calculations nicely verify that our experimental results are order-of-magnitude correct.

Supplementary Note 2: Supplementary Figures

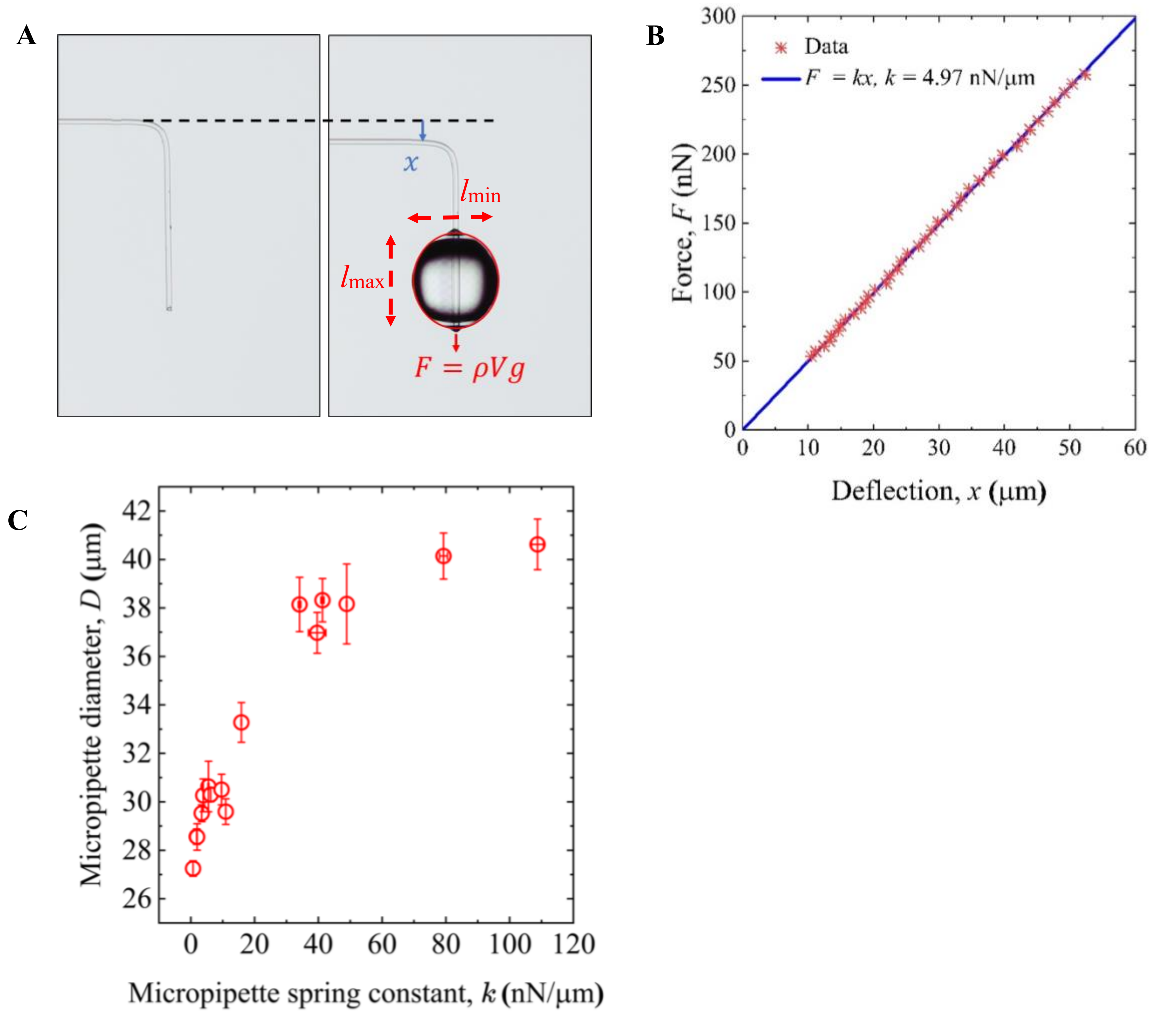

Supp. Fig. S1

Quasi-static calibration. A) The displacement x of the MFS cantilever under the weight of a water droplet that is injected through a syringe. The droplet size is increased linearly with time and the corresponding deflection is recorded with time. The weight of the droplet is calculated from the volume, V and density, ρ. The droplet is modelled as an ellipsoid (red outline) using image analysis in MATLAB, and the volume is calculated as $V = \pi l_{\min}^2 l_{\max}/6$ with a minimum and maximum diameter, $l_{\min}$ and $l_{\max}$, respectively. **B)** The force exerted, that is, the weight of the water droplet ($F = \rho V g$), is directly proportional to the displacement x. The slope in the graph gives the spring constant (k) of the micropipette. **C)** Micropipette tip diameter as a function of spring constant. The error bars are standard deviations from several measurements.

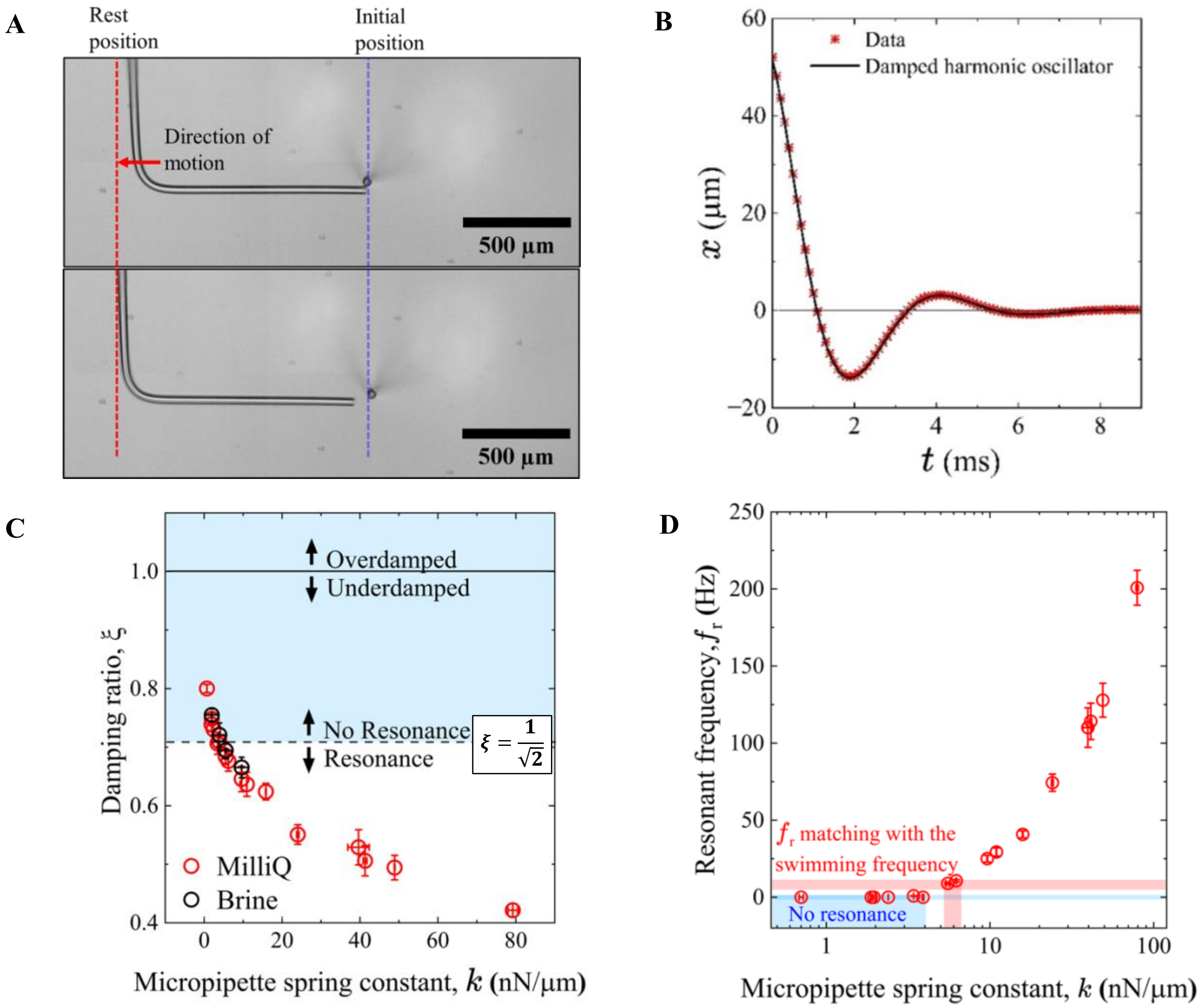

Supp. Fig. S2

Dynamic calibration. A) The dynamic calibration experiment is carried out by deflecting the MFS in a petri dish containing milliQ water. A stiff L-shaped micropipette, positioned orthogonally to the MFS, is used as a probe for deflection. The experiments are carried out for displacements range of ca. 50 to 450 μm. **B)** The damped harmonic oscillation model is fit to the experimental time-deflection data to obtain the parameters: undamped angular frequency ω_o, damping ratio ξ, initial amplitude A, and phase φ. **C.** The damping ratio ξ decreases with spring constant k. For $\xi \leq 1/\sqrt{2}$, there is no resonance effect. The value of ξ determine the behaviour of the system to be overdamped ($\xi >$ 1), underdamped ($\xi < 1$), or critically damped ($\xi = 1$). If $\xi > 1$ then the system exponentially decays to steady state without oscillating. The larger the value, the slower the system reaches equilibrium. If $\xi = 1$, the system returns to steady state quickly without oscillating. If $\xi < 1$, the system oscillates with slight small frequency and gradually decreases to zero. The results are the same in milliQ water and brine solution (in which the *Artemia* swimming experiments were performed). **D)** For MFS with $k < 4$ nN/μm, marked by the blue region, there is no resonant frequency. The red region indicates resonant frequencies that match with the swimming frequency of *Artemia* (5–10 Hz). Therefore, the micropipettes within this resonant frequency regime are not used for measuring swimming forces of *Artemia*. The error bars for ξ and f_r in C and D are propagated using the 95% confidence interval of the fitting parameters, and the error bar for k is the standard deviation from several calibration experiments.

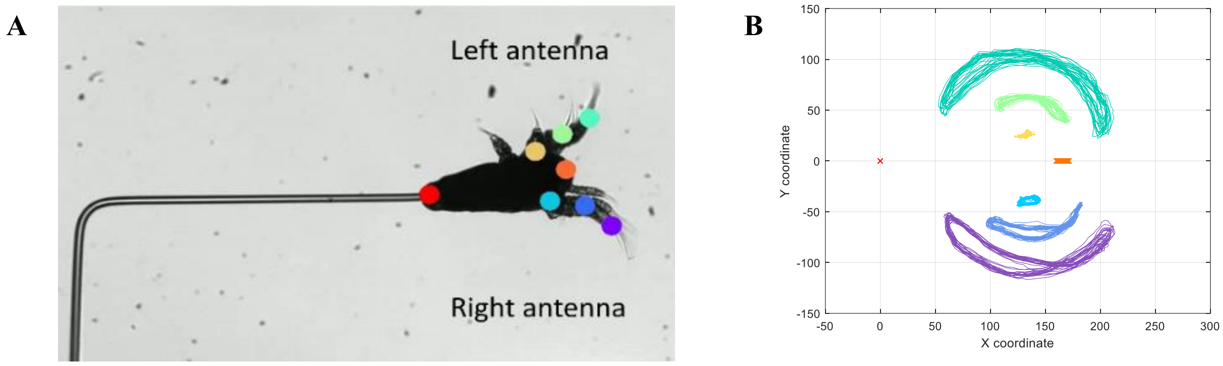

Supp. Fig. S3.
DeepLabCut motion tracking of swimming *Artemia*. A) Markers created by DeepLabCut for a tethered swimmer. **B)** Trajectories of the markers plotted in the *xy*-plane of the swimmer.

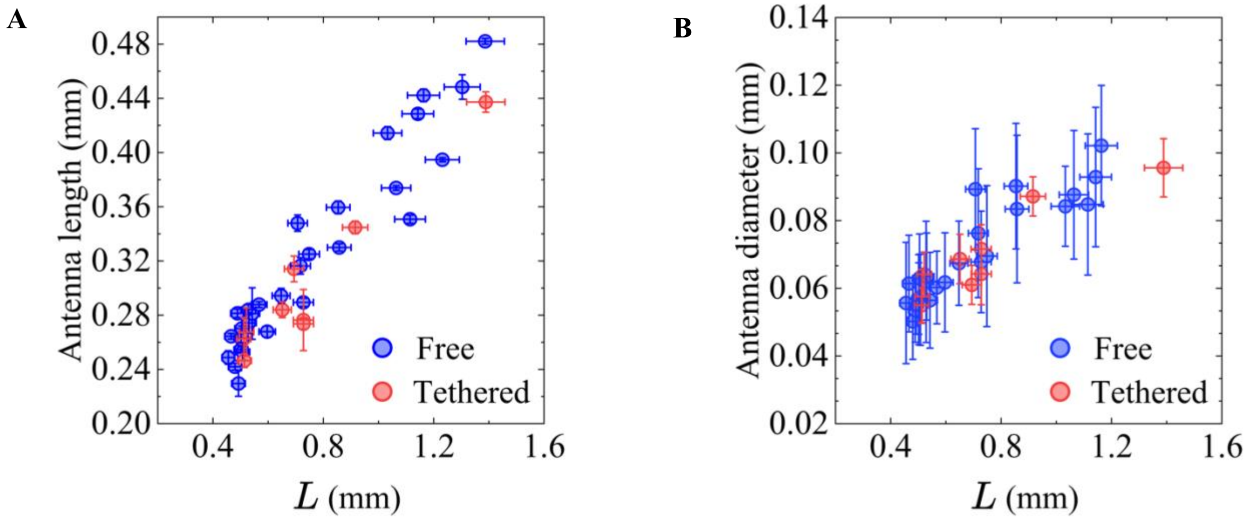

Supp. Fig. S4.
Antenna dimensions. A) The antenna length and **B)** diameter increase as the *Artemia* grows. The error bars are standard deviations from several measurements.

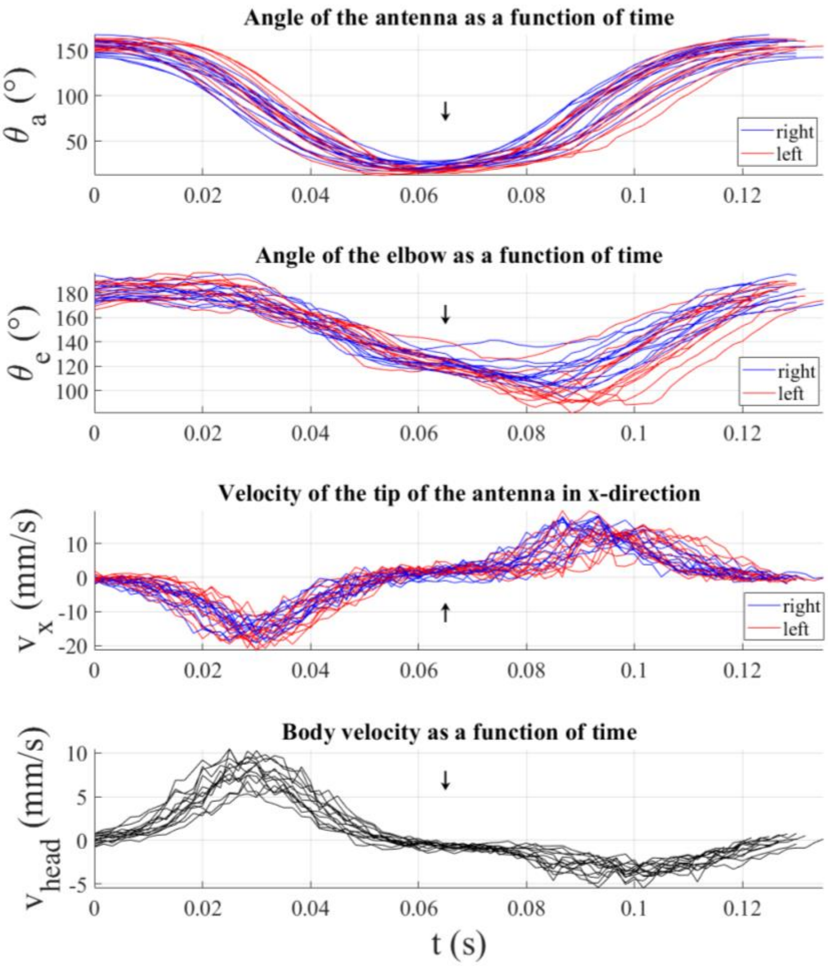

Supp. Fig. S5A

***Artemia* free-swimming kinematics.** The left and right "armpit" angle θ_a, "elbow" angle θ_e, and antenna tip velocity (see definitions in **Fig. 3A**), as well as the head velocity over several swimming cycles for an *Artemia* with a body length of 441 µm. The arrow indicates the start of the recovery-stroke.

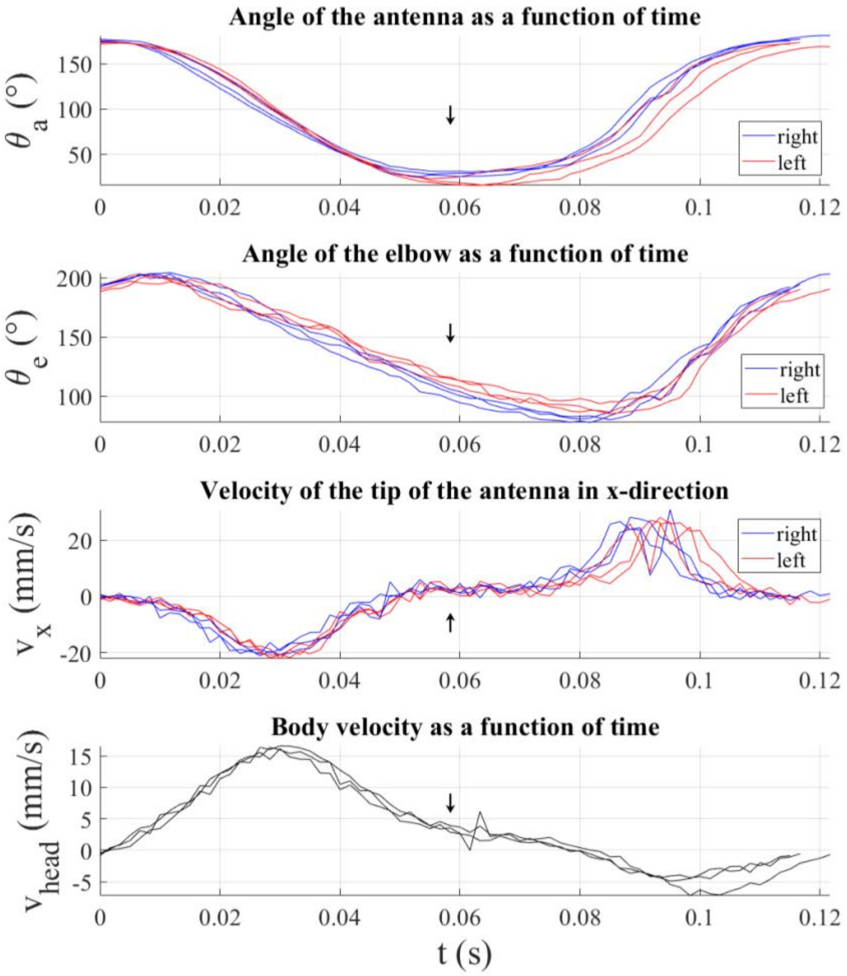

Supp. Fig. S5B

***Artemia* free-swimming kinematics.** The left and right "armpit" angle θ_a, "elbow" angle θ_e, and antenna tip velocity (see definitions in **Fig. 3A**), as well as the head velocity over several swimming cycles for an *Artemia* with a body length of 735 µm. The arrow indicates the start of the recovery-stroke.

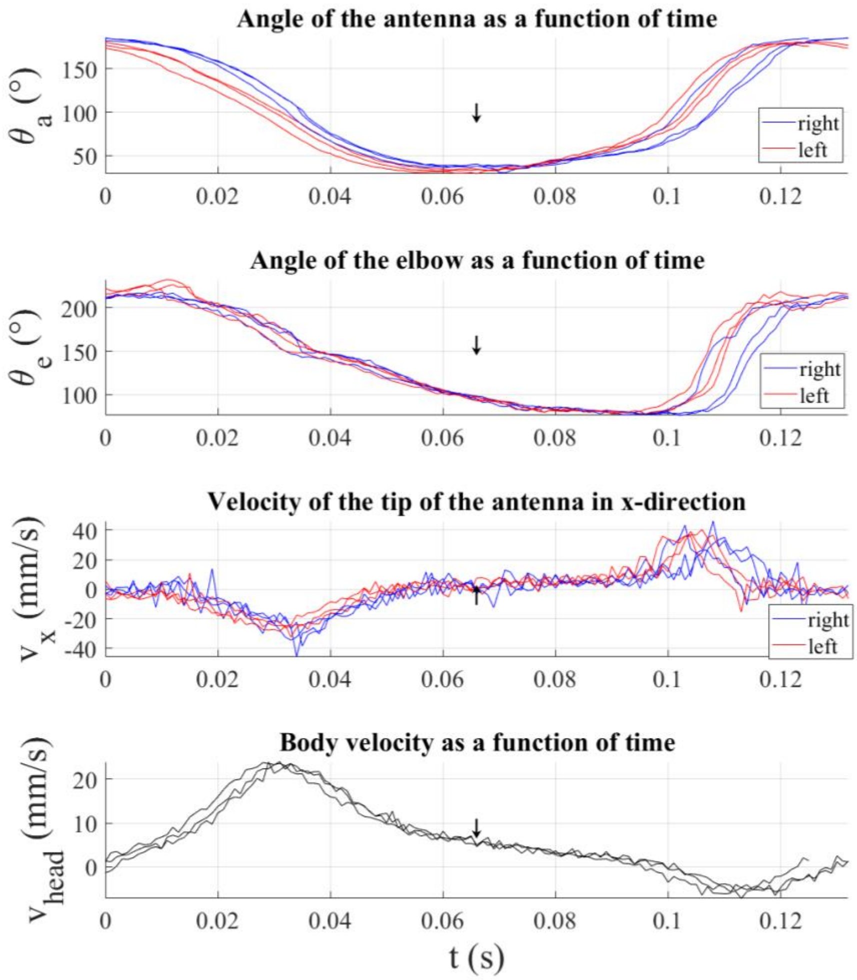

Supp. Fig. S5C

***Artemia* free-swimming kinematics.** The left and right "armpit" angle θ_a, "elbow" angle θ_e, and antenna tip velocity (see definitions in **Fig. 3A**), as well as the head velocity over several swimming cycles for an *Artemia* with a body length of 1159 µm. The arrow indicates the start of the recovery-stroke.

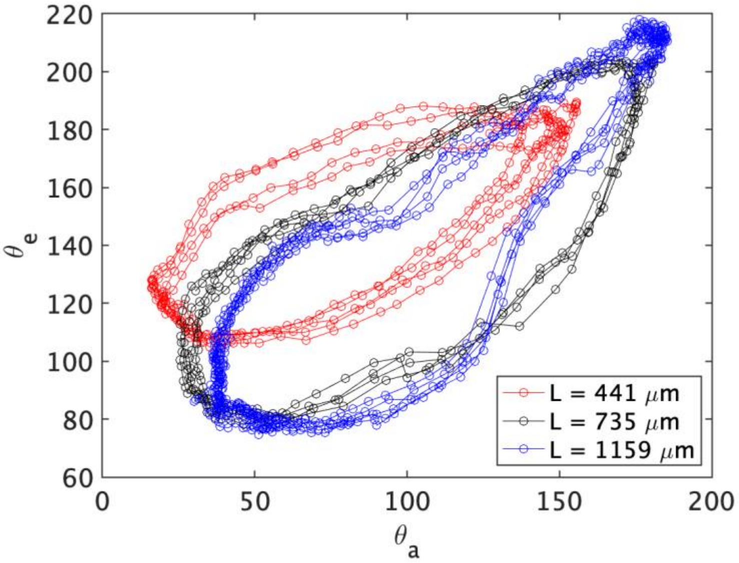

Supp. Fig. S5D

***Artemia* free-swimming kinematics.** The "elbow" angle θ_e plotted against the "armpit" angle θ_a for several swimming cycles with three differently sized, free-swimming *Artemia* (same as in A–C).

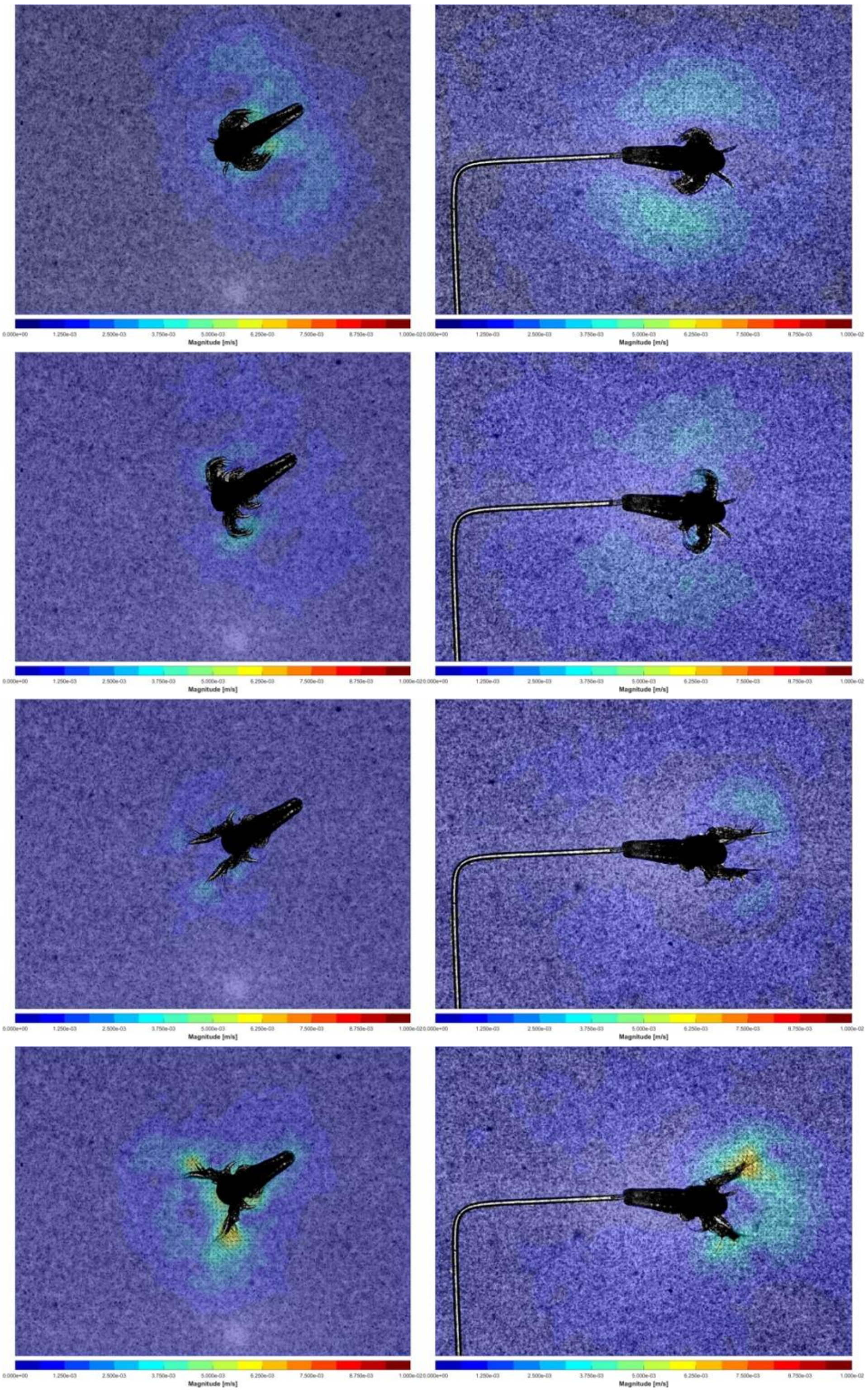
0.000e+00
1.250e-03
2.500e-03
3.750e-03
5.000e-03
6.250e-03
7.500e-03
8.750e-03
1.000e-02
Magnitude [m/s]

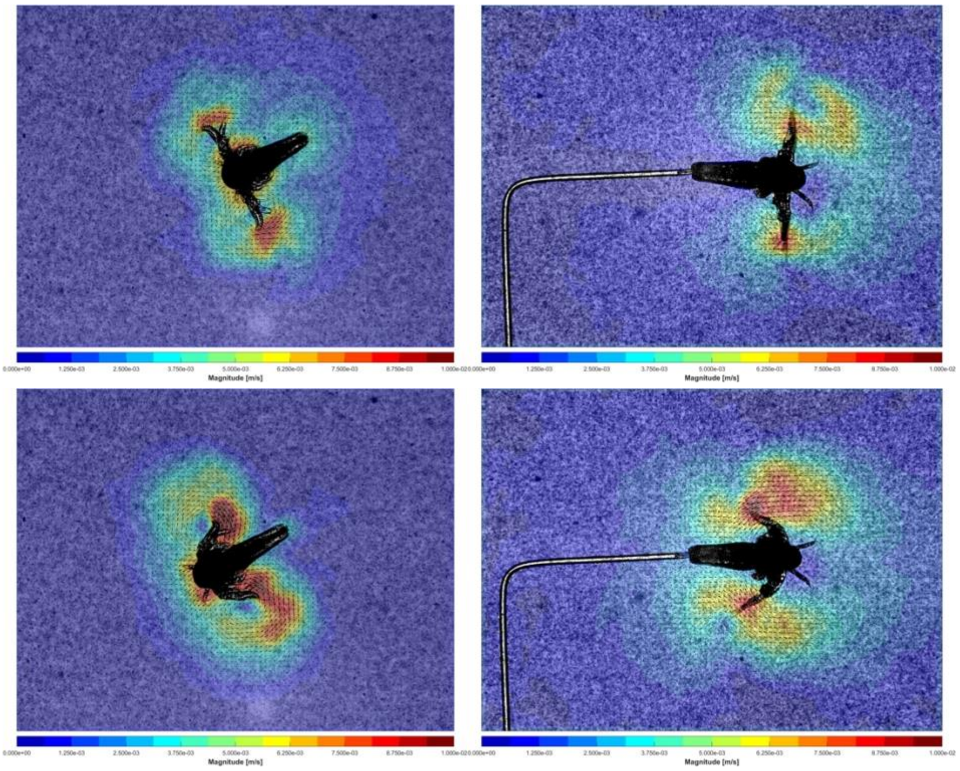

Supp. Fig. S6.

Fluid flow. Comparative analysis of the flow fields between free-swimming (left column) and tethered (right column) *Artemia* using particle image velocimetry (PIV). The snapshots are from a similarly sized *Artemia* from similar positions in the swimming cycle (length and velocity scale same in all images). The magnitudes of the flow fields around the propelling antenna are quantitatively very similar between the two experiments. The main difference comes in the fluid flow around the body, where the free swimmer pushes fluid forward while moving whereas the fluid remains stationary around the head of the tethered swimmer. The most significant physical aspect of swimming depends on the actively moving limbs (*25*), while the passive drag on the body is not of as great importance. To test for this, tethered experiments were performed with cantilevers of different spring constants, where the softer MFSs allow for more back and forth motion of the body in the fluid, mimicking free swimming a bit better. Within experimental error, there is no difference in the measured propulsive forces depending on the MFS spring constant (**Fig. S7B**).

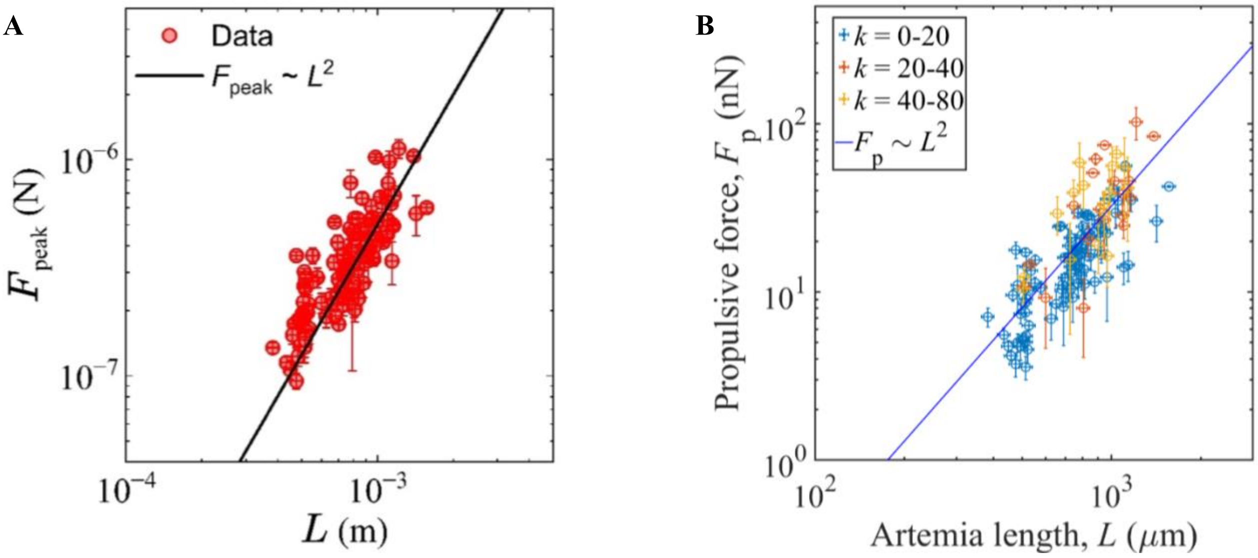

Supp. Fig. S7.

Propulsive forces. A) The peak-to-peak force as a function of body length as measured for 129 individuals of different age. **B)** The mean propulsive force as a function of body length with the MFS spring constant highlighted in different colours. The measured propulsive force is not dependent on the magnitude of the spring constant used.

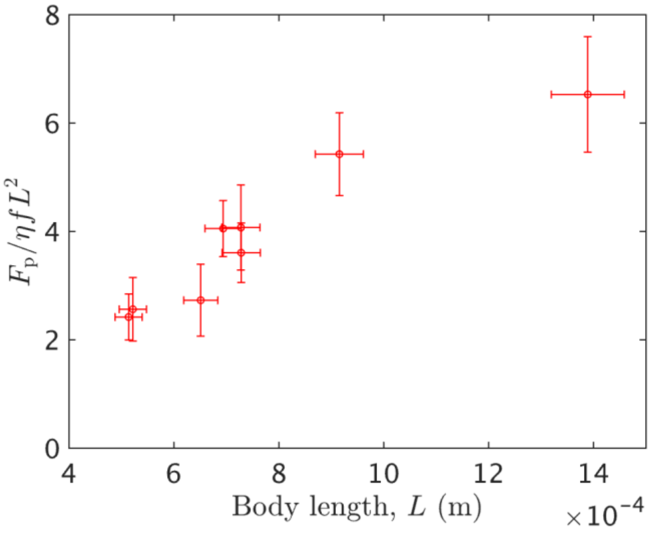

Supp. Fig. S8.

Normalised propulsive force. The mean propulsive force normalised according to the Stokes' regime model plotted as a function of body length. The larger swimmers have a factor of ca. 3 higher normalised swimming forces than the smaller swimmers, showing that the model does not successfully describe the swimming dynamic of *Artemia*.

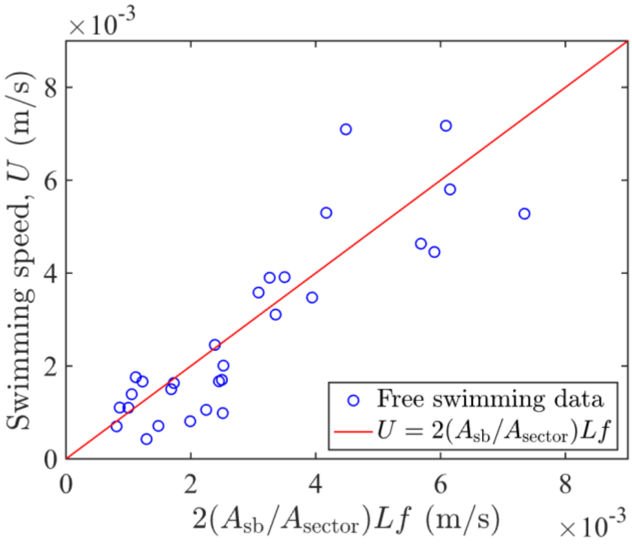

Supp. Fig. S9
Free-swimming speed. Mean swimming speed of free *Artemia* as a function of body length.

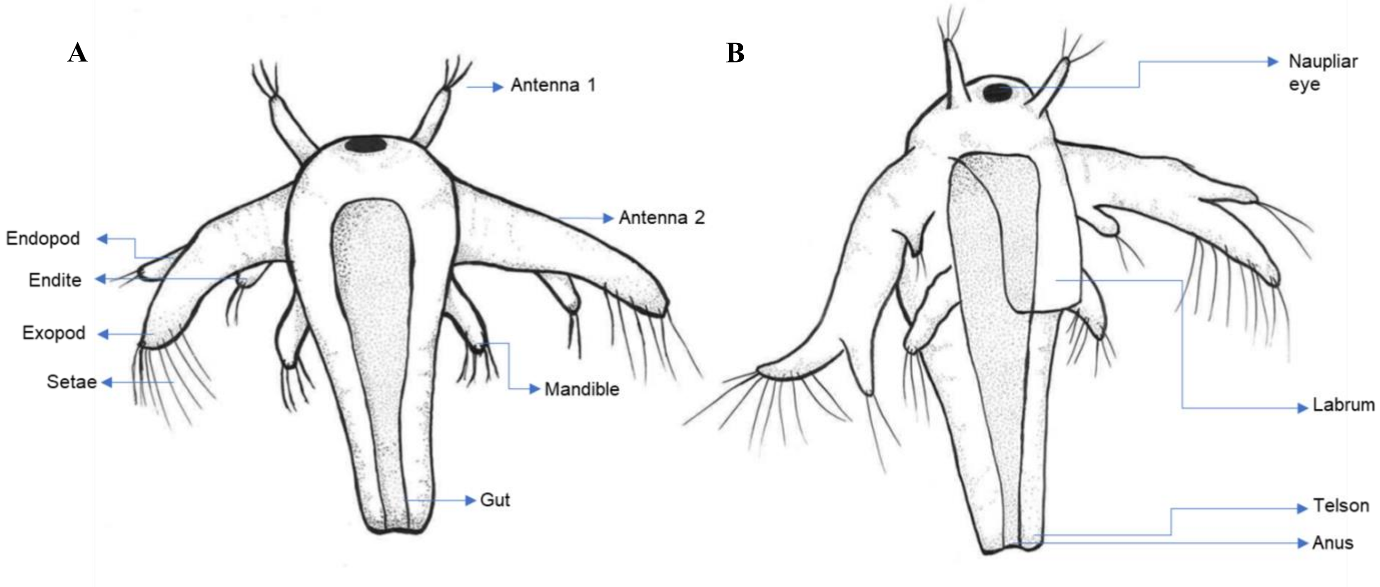

Supp. Fig. S10
Anatomy of *Artemia* larval stages used in experimental procedures. Schematics drawing of Instar I (newly hatched nauplius) with **A)** dorsal and **B)** ventral view. The secondary antennae are biramous and comprised of an exopod (or main branch), endopod (or secondary ramification) as well as an endite (an accessory structure used for feeding and sensing). The exopod, the main and larger branch, is lined with evenly spaced setae that vary in number and size as the organism grows. The endopod is smaller, and the presence of setae is species-dependent, with some displaying fewer and/or smaller setae. The endite, a lobe-like structure, plays a role in feeding and has sensory functions.

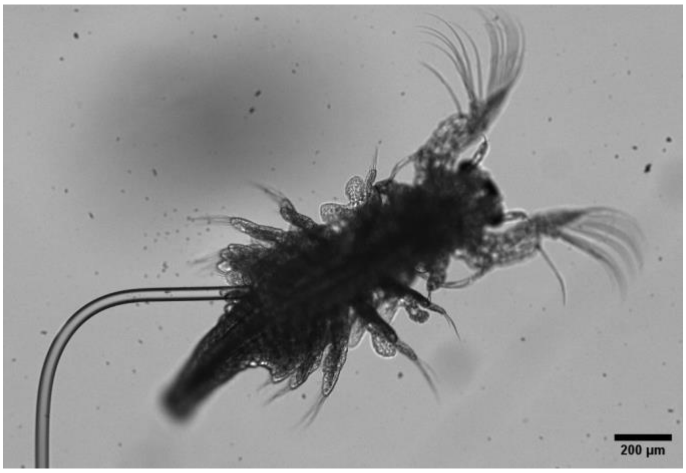

Supp. Fig. S11
Adult *Artemia*. Image of an adult *Artemia* swimming with its thoracopods.

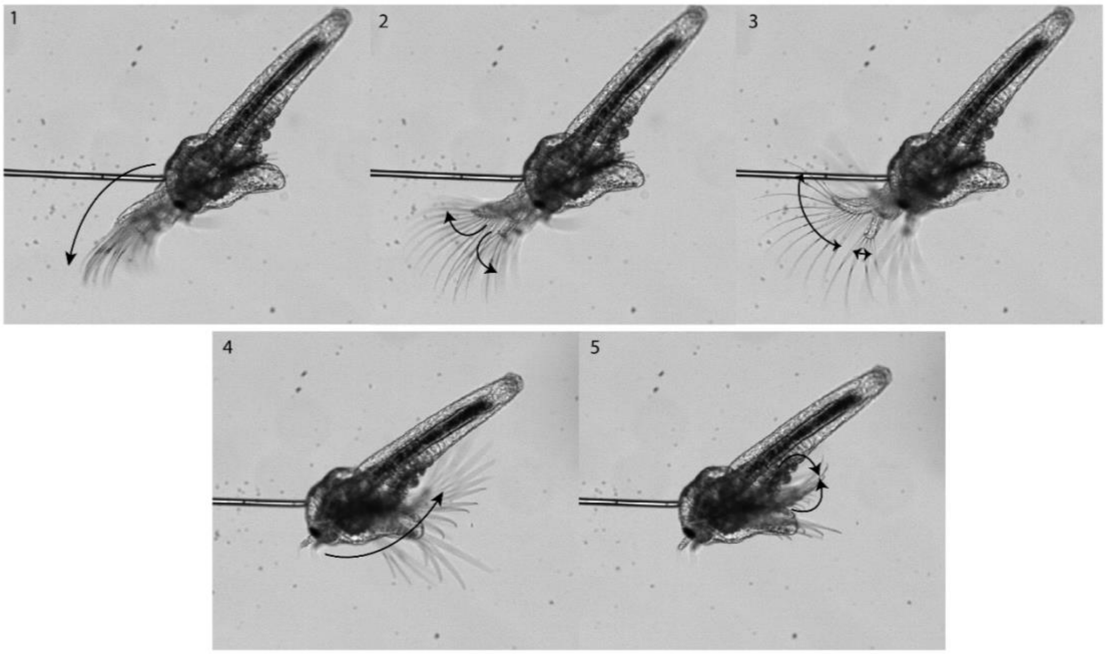

Supp. Fig. S12

Secondary antenna stroke behaviour. Side view of the progression and behaviour of the exopod, endopod and their associated setae during a swimming stroke. **1)** The exopod is brought forward with flexed joints and with the setae contracted in a bundle-like formation. **2)** The exopod is fully extended and the setae bundle begins to separate. **3)** Both the exopod and the endopod separate and their associated setae are fully spread in a fan-like display. **4)** The artemia executes the power stroke with fully extended appendages. **5)** At the end of the power stroke the exopod, endopod and their setae are contracted into the bundle-like formation and the antennae start to move forward as described in the first step.

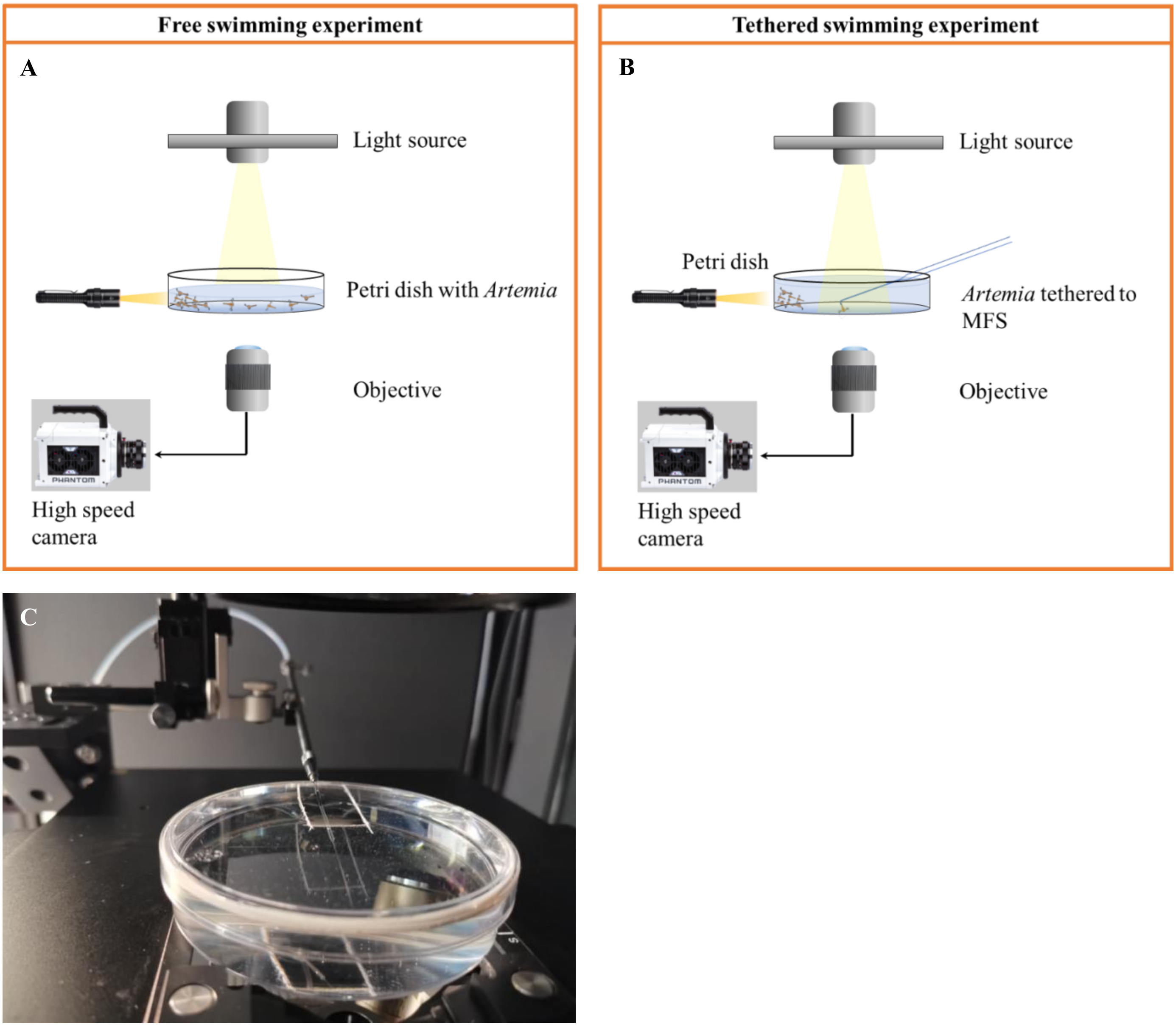

Supp. Fig. S13
The experimental setups. Schematic drawings of the **A)** free-swimming and **B)** tethered swimming setups. **C)** Photo of the tethered experimental setup.

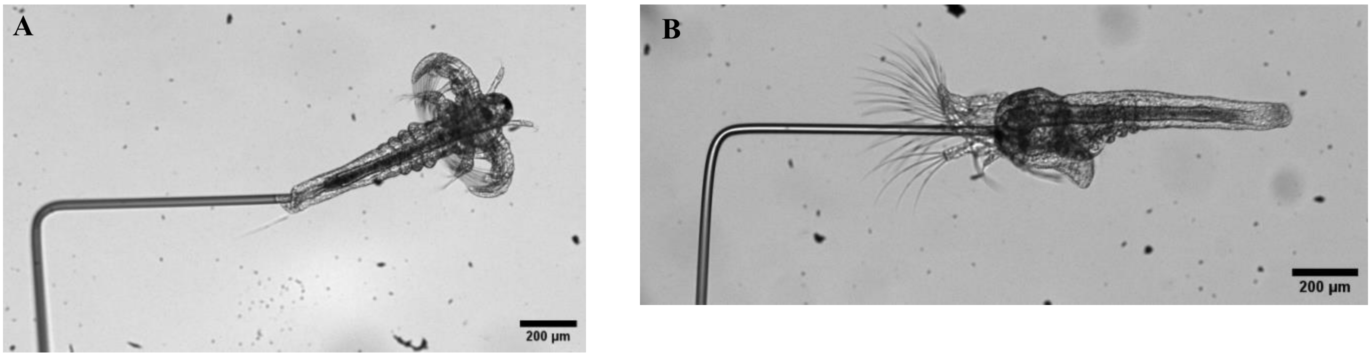

Supp. Fig. S14

Examples of bad MFS catches. Images of **A)** angled and **B)** rotated *Artemia* caught with an MFS. The force data sets from these types of experiments were discarded.
