# Supplementary material for: Forces and symmetry breaking of a living meso-swimmer
Source: Commun Phys. 2026 Jan 8;9(1):53. doi: 10.1038/s42005-025-02486-3 (PMC12893912; doi:10.1038/s42005-025-02486-3)
Supplement: Supplementary file 2 — Description of Additional Supplementary Files [file 42005_2025_2486_MOESM2_ESM.pdf]

Description of Additional Supplementary Files

File name- Supplementary Movie 1

File description-Swimming force measurement on Artemia. High-speed movie from MFS swimming force experiment on Artemia (length L = 520 ± 30 μm, same as in Fig. 2A) caught by the head through suction. During the force experiment, a high-speed camera captured the pipette deflection as well as the motion of the shrimp body during several swimming cycles.

File name- Supplementary Movie 2

File description-Dynamic MFS calibration. High-speed movie from a dynamic MFS calibration experiment. The pipette is deflected from its equilibrium position (not shown in movie) and released to move as a damped harmonic oscillator.

File name-Supplementary Movie 3

File description-Tethered swimming of adult Artemia. High-speed movie from an unsuccessful MFS swimming force experiment on an adult Artemia.

File name-Supplementary Movie 4

File description-Advanced image analysis of tethered Artemia kinematics. High-speed movie from MFS swimming force experiment on Artemia analysed with the deep neural network-based software DeepLabCut to track the motion of 8 different body parts as a function of time.

File name- Supplementary Movie 5

File description-Advanced image analysis of free-swimming. Artemia kinematics. High-speed movie from free-swimming experiment on Artemia analysed with the deep neural network-based software DeepLabCut to track the motion of 8 different body parts as a function of time.
