# Supplementary material for: Forces and symmetry breaking of a living meso-swimmer
Source: Commun Phys. 2026 Jan 8;9(1):53. doi: 10.1038/s42005-025-02486-3 (PMC12893912; doi:10.1038/s42005-025-02486-3)
Supplement: Supplementary file 8 — Reporting Summary [file 42005_2025_2486_MOESM8_ESM.pdf]

nature portfolio

Corresponding author(s): Matilda Backholm

Last updated by author(s): 7.8.2025

Reporting Summary

Nature Portfolio wishes to improve the reproducibility of the work that we publish. This form provides structure for consistency and transparency in reporting. For further information on Nature Portfolio policies, see our Editorial Policies and the Editorial Policy Checklist.

Statistics

For all statistical analyses, confirm that the following items are present in the figure legend, table legend, main text, or Methods section.

n/a	Confirmed	
☐	☒	The exact sample size (n) for each experimental group/condition, given as a discrete number and unit of measurement
☐	☒	A statement on whether measurements were taken from distinct samples or whether the same sample was measured repeatedly
☒	☐	The statistical test(s) used AND whether they are one- or two-sided<br>*Only common tests should be described solely by name; describe more complex techniques in the Methods section.*
☒	☐	A description of all covariates tested
☒	☐	A description of any assumptions or corrections, such as tests of normality and adjustment for multiple comparisons
☒	☐	A full description of the statistical parameters including central tendency (e.g. means) or other basic estimates (e.g. regression coefficient) AND variation (e.g. standard deviation) or associated estimates of uncertainty (e.g. confidence intervals)
☒	☐	For null hypothesis testing, the test statistic (e.g. F, t, r) with confidence intervals, effect sizes, degrees of freedom and P value noted<br>*Give P values as exact values whenever suitable.*
☒	☐	For Bayesian analysis, information on the choice of priors and Markov chain Monte Carlo settings
☒	☐	For hierarchical and complex designs, identification of the appropriate level for tests and full reporting of outcomes
☒	☐	Estimates of effect sizes (e.g. Cohen's d, Pearson's r), indicating how they were calculated

Our web collection on statistics for biologists contains articles on many of the points above.

Software and code

Policy information about availability of computer code

Data collection	Data collection was conducted using commercial software, Phantom Camera Control (PCC) from Vision Research Inc.
Data analysis	Data analysis and graphical representation were conducted using MATLAB. For data analysis, the code employed was adapted from Backholm (2019).

For manuscripts utilizing custom algorithms or software that are central to the research but not yet described in published literature, software must be made available to editors and reviewers. We strongly encourage code deposition in a community repository (e.g. GitHub). See the Nature Portfolio guidelines for submitting code & software for further information.

Data

Policy information about availability of data

All manuscripts must include a data availability statement. This statement should provide the following information, where applicable:

- Accession codes, unique identifiers, or web links for publicly available datasets
- A description of any restrictions on data availability
- For clinical datasets or third party data, please ensure that the statement adheres to our policy

The datasets used for plotting all graphs in the paper, examples of raw data files, and the new MATLAB analysis codes written for this manuscript will be shared on Zenodo when the manuscript has been accepted for publication. ¨R. A. Lara, N. Sharadhi, A. A. L. Huttunen, L. Ansas, E. J. G. Rislakki, G. M. Bessa, and M. Backholm, Data from "Forces and symmetry breaking of a living meso-swimmer," Zenodo (2025); https://doi.org/10.5281/zenodo.14513461.¨

Research involving human participants, their data, or biological material

Policy information about studies with human participants or human data. See also policy information about sex, gender (identity/presentation), and sexual orientation and race, ethnicity and racism.

Reporting on sex and gender	N/A
Reporting on race, ethnicity, or other socially relevant groupings	N/A
Population characteristics	N/A
Recruitment	N/A
Ethics oversight	N/A

Note that full information on the approval of the study protocol must also be provided in the manuscript.

Field-specific reporting

Please select the one below that is the best fit for your research. If you are not sure, read the appropriate sections before making your selection.

☒ Life sciences ☐ Behavioural & social sciences ☐ Ecological, evolutionary & environmental sciences

For a reference copy of the document with all sections, see nature.com/documents/nr-reporting-summary-flat.pdf

Life sciences study design

All studies must disclose on these points even when the disclosure is negative.

Sample size	The final sample size was determined after collecting a sufficient number of data points to span the length range corresponding to the naupliar stages of our experimental model prior to the onset of the next developmental stage and its associated significant morphological modifications. This enabled and accurate representation of all naupliar stages, allowing for a comprehensive morphological characterization and robust biological interpretations.
Data exclusions	Individuals displaying any degree of morphological abnormalities or anatomical malformations were excluded from the final dataset. Additionally, for tethered swimmers, specimens captured at an angle relative to the cantilever or tilted with respect to the focal plane were omitted as well.
Replication	The methodology has been clearly outlined in its section, with references to relevant published literature and detailed explanations of the modifications and implementations derived from these sources. This methodology was consistently applied throughout the entire data acquisition period, ensuring high reproducibility already within the scope of this manuscript and supporting future replication efforts.
Randomization	Organisms sampling was randomized through a multi-step process. First, experimental individuals were obtained from batches containing thousands of individuals at varying hatching times and therefore sizes. Second, experimental arenas were filled with aliquots of hundreds of randomly selected individuals from these batches. Third, for tethered experiments, selection bias was inherently minimized due to the erratic mobility of the organisms; individuals were captured at illuminated spots designed to attract a swarm, making targeted selection impossible.
Blinding	Blinding was not required in this study, as the experimental animals were not subjected to any form of treatment.

Reporting for specific materials, systems and methods

We require information from authors about some types of materials, experimental systems and methods used in many studies. Here, indicate whether each material, system or method listed is relevant to your study. If you are not sure if a list item applies to your research, read the appropriate section before selecting a response.

Materials & experimental systems

n/a	Involved in the study	
☒	☐	Antibodies
☒	☐	Eukaryotic cell lines
☒	☐	Palaeontology and archaeology
☐	☒	Animals and other organisms
☒	☐	Clinical data
☒	☐	Dual use research of concern
☒	☐	Plants

Methods

n/a	Involved in the study	
☒	☐	ChIP-seq
☒	☐	Flow cytometry
☒	☐	MRI-based neuroimaging

Animals and other research organisms

Policy information about studies involving animals; ARRIVE guidelines recommended for reporting animal research, and Sex and Gender in Research

Laboratory animals	In this study, we used commercially available Artemia sp. cysts (Crustacean, Anostraca), which were hatched under controlled conditions in our laboratory.
Wild animals	N/A
Reporting on sex	N/A
Field-collected samples	N/A
Ethics oversight	Research on Artemia sp. is currently exempt from the application of ethical authorizations. Due to the absence of a notochord, these organisms lack the neural complexity for a subjective sense of pain.

Note that full information on the approval of the study protocol must also be provided in the manuscript.

Plants

Seed stocks	N/A
Novel plant genotypes	N/A
Authentication	N/A
